# The computational approaches of lncRNA identification based on coding potential: *Status quo* and challenges

**DOI:** 10.1016/j.csbj.2020.11.030

**Published:** 2020-11-19

**Authors:** Jing Li, Xuan Zhang, Changning Liu

**Affiliations:** a CAS Key Laboratory of Tropical Plant Resources and Sustainable Use, Xishuangbanna Tropical Botanical Garden, Chinese Academy of Sciences, Menglun, Mengla, Yunnan 666303, China; b Center of Economic Botany, Core Botanical Gardens, Chinese Academy of Sciences, Menglun, Mengla, Yunnan 666303, China; c The Innovative Academy of Seed Design, Chinese Academy of Sciences, Menglun, Mengla, Yunnan 666303, China

**Keywords:** LncRNA identification, *In sillico*, Algorithm, Feature, Coding potential, sORF

## Abstract

Long noncoding RNAs (lncRNAs) make up a large proportion of transcriptome in eukaryotes, and have been revealed with many regulatory functions in various biological processes. When studying lncRNAs, the first step is to accurately and specifically distinguish them from the colossal transcriptome data with complicated composition, which contains mRNAs, lncRNAs, small RNAs and their primary transcripts. In the face of such a huge and progressively expanding transcriptome data, the *in-silico* approaches provide a practicable scheme for effectively and rapidly filtering out lncRNA targets, using machine learning and probability statistics. In this review, we mainly discussed the characteristics of algorithms and features on currently developed approaches. We also outlined the traits of some state-of-the-art tools for ease of operation. Finally, we pointed out the underlying challenges in lncRNA identification with the advent of new experimental data.

## 1 Introduction

Over the past two decades, the development of high-throughput RNA-sequencing technologies have revealed that the vast majority of eukaryotic genomes is transcribed into non-protein coding RNAs (ncRNAs) [109], [21], [69], [75], [29], [55]. By far, there are more than 229 public ncRNA databases, which are divergent according to information source, type of RNA, source organisms, data formats, and the mechanisms for information retrieval [93]. Among all of the ncRNAs, long noncoding RNAs (lncRNAs) – transcripts of length above 200nt -- have aroused intense interests due to their significant roles in many biological processes and diseases, such as epigenetic modification, gene and protein expression regulation, and cancer progression [15], [44], [113], [118], [121], [140]. Different tools have been developed to identify lncRNAs, predict their function and correlate with various diseases [10], [100], [2]. Many lncRNAs share similar features with classical mRNAs, such as transcription by polymerase II with a 5′-cap and 3′-polyadenylated tail, splicing pattern, sequence length, frequent accumulation in the cytoplasm, and even overlap with coding genes [135], [136], [96], [117], [151]. Therefore, when facing the rapidly augmented transcriptome data, the primary challenge is how to effectively distinguish long non-coding transcripts from protein-coding genes, especially for those *de novo* transcriptome assembly in the absence of highly confident reference genome.

Machine-learning-based *in-silico* methods provide a viable approach for efficiently and rapidly identifying lncRNAs. In recent years, a plenty variety of computational methods have been developed based on the substantial amount of publicly-available transcriptome data and databases. These approaches typically apply various algorithm models to screen out noncoding from coding, by integrating the differentiated features between lncRNAs and mRNAs. The earliest classification tools, such as CONC (Coding Or Non-Coding) and CPC (Coding Potential Calculator), focused on the coding capability of transcript itself, and much rely on the recorded coding gene databases [86], [72]. However, with the accumulated number of lncRNAs found in diversified species, the intrinsic distinctive features of lncRNAs are further assessed and weighted in characterization of RNA coding potential. Until now, many features are integrated into lncRNA’s identification, including ORF length and coverage, nucleotide composition and codon usage, conservation scores, k-mer sequence, RNA secondary structure, ribosome release score (RRS) and etc [72], [1], [80], [84], [139], [141], [130], [57]). For example, CPAT used logistic regression model by integrating four features (ORF length, ORF coverage, Fickett score and Hexamer usage preference) [141]; CNCI used support vector machine (SVM) and hexamer for distinguishing ncRNAs from coding RNAs [130]; PLEK applied a SVM algorithm based on an improved k-mer scheme [80]; FEELnc exploited random forest algorithm by extracting features of ORF coverage, codon usage and nucleotide frequency [146].

On the other hand, with the development of recognition for lncRNAs, we start to re-examine the “coding” concept of RNAs. The results of advanced ribosome profiling have revealed that a considerably large part of lncRNAs tend to contain short open reading frames (sORFs) and bind with ribosomes [63], [7], [120], [4], [90], [82], [25]. Moreover, increasing evident showed that these noncoding transcripts are capable of encoding functional micropetides (<=100 amino acids, AAs) [59], [82], [53], [152]. These micropeptide functions are not exclusive against noncoding function, but mutually compatible with each other. All these facts raise questions on the fitness of current binary classification on RNAs, and how should we deal with new data when new information is provided. In this review, we summarized the current *in-silico* methods on lncRNA’s identification and outlined their individual traits. We also discussed the underlying challenges when facing new data on this field.

## 2 General profile for lncRNA identification tools

In the beginning of the 21st century, as more attentions were paid on lncRNAs which constitute the majority of noncoding transcripts [114], the development of highly-resolvable *in-silico* approaches to extract the lncRNA components from the huge number of transcriptome data is on an urgent demand. Up to date, there have been dozens of tools being developed for lncRNA identification. For each method, the key signatures are algorithm model and selected features. Thus, we outlined the algorithm models and features of present computational tools in Table 1 (see Supplementary Table S1 for more details).

**Table 1:** The algorithm models and feature extraction of present computational approaches.

Tools	Year	Algorithm Model	Features							Reference
			1st sequence-Related	2nd Structure-Related	Phylogenetic-conservation	Exprimental -Related	Translation-Related	PhysiChemi-Related	Combined /Transformed features	
CONC	2006	SVM	√	√					√	[86]
CPC	2007	SVM	√							[72]
PORTRAIT	2009	SVM	√					√	√	[6]
PhyloCSF	2011	Continuous-time Markov processes	√		√					[84]
CPAT	2013	Logistic regression	√							[141]
CNCI	2013	SVM	√			,				[130]
iSeeRNA	2013	SVM	√							[128]
Linc-SF	2013	SVM	√	√			√			[142]
PLEK	2014	SVM	√							[80]
LncRNA-ID	2015	Random Forest	√				√			[1]
LncRNA-MFDL	2015	Deep learning	√	√			√			[37]
LncRScan-SVM	2015	SVM	√				√			[129]
DeepLNC	2016	Deep learning	√							[134]
COME	2016	BRF	√	√		√				[57]
lncScore	2016	Logistic regression model	√				√			[154]
Lncident	2016	SVM	√							[49]
LncRNApred	2016	Random forest	√						√	[111]
longdist	2017	SVM	√							[123]
CPC2	2017	SVM	√					√		[68]
FEELnc	2017	Random Forest model	√						√	[146]
PLncPRO	2017	Random forest	√						√	[126]
PlantRNA_Sniffer	2017	SVM	√							[138]
TLCLnc	2017	Ensembled two-layer structured classifier	√						√	[56]
LncADeep	2018	Deep learning	√						√	[148]
BASiNET	2018	Graph network	√						√	[64]
CREMA	2018	Ensemble machine learning classifiers	√							[125]
TERIUS	2018	Kernel density estimation	√			√	√			[26]
lncRNAnet	2018	Deep learning	√							[8]
IRSOM	2018	Deep neutral network	√							[112]
LncFinder	2019	Logistic regression, SVM, Random forest, ELM, Deep learning	√	√				√		[50]
CPPred	2019	SVM	√				√	√		[133]
LGC	2019	Maximum Likelihood Estimation	√						√	[139]
PLIT	2019	Random Forest	√						√	[32]
lncRNA-LSTM	2019	Deep learning method	√							[98]
LncPred-IEL	2019	Ensemble machine learning classifiers	√				√	√	√	[147]
RNAplonc	2019	Eight machine learning algorithms	√	√			√			[108]
PredLnc-GFStack	2019	Stacked Ensemble Learning	√				√	√		[87]
CNIT	2019	XGBoost	√				√			[46]
CodAn	2020	GHMMs	√							[101]
NCResNet	2020	Deep learning	√				√	√		[149]

### 2.1 Algorithm models used in present computational tools

One pivotal step of machine learning is to explore the intrinsic characteristics from huge and complex data for classification, which requires the reliable algorithm models to support. Up to now, many efficient algorithm models are implemented in lncRNA identification, including logistic regression, SVM, random forest (RF), and deep learning algorithm, etc. Wherein, SVM algorithm, a classifier based on hyperplane and kernel function, was widely adopted due to its stability and availability [72], [130], [80], [57]. SVM can use kernel functions to increase the dimension of the space so as to extremely separate sets of data by constructing a separating margin or hyperplane at higher dimensions [30]. The data points that can be used to determine the hyperplane are called support vectors. There are several ready-made libraries for SVM, such as libSVM [22], which greatly promoted the implementation of SVM. Up to now, more than a dozen of tools had adopted SVM as algorithm model, like CPC, CNCI, PLEK, COME, CPPred, etc.

RF model is an optimized version of decision-tree model by bagging, which randomly and repeatedly extracts samples from the whole data for training and uses average values as output [54]. This model could greatly avoid the bad sample (noise) and thus improve the accuracy. It can integrate multidimensional features as well as evaluate the weights of different features. During the training process, the interaction between features can be detected. For unbalanced data sets, it can balance the errors; hence, if a large percentage of features are missing, accuracy can still be maintained. However, RF models have been demonstrated to be overfitting in some classification or regression problems, when the noise is too much. Besides, for the data that may have many features with polarized weight values, the more weight value the greater impact on the random forest, which possibly leads to the incredibility of results of classification under such weight assumption [1], [146]. There are a few tools employing RF as model, such as LncRNA-ID, FEELnc, etc [1], [146]).

Deep learning is a state-of-the-art classification algorithm thrived in recent years, by which computer can automatically learn the pattern characteristics and integrate them into model establishment [77]. Deep learning concept rooted from artificial neural network research, which are composed of three basic layers (input layer, hidden layer, output layer), and imitate human brain to explain the mechanism of data. The word “deep” in deep learning refers to the use of multiple layers through which the data is transformed. With the emergence of deep learning, we do not need to do a lot of feature engineering, such as designing the content of features or the combination of features and so on. But deep learning has a relatively high requirement on data size, and is involved with some complicated modulation procedure, such as hyperparameter tuning, regularization and optimization.[37], [134], [148]. In addition, the process of a deep neural network operation likes a black box, from which it is hard and difficult to interpret the performance and evaluate the importance of every input feature [149]. Such methods include LncRNA-MFDL, DeepLNC, LNCAdeep, NCResNet and so on [37], [134], [148], [149].

Moreover, in order to enhance performances, several ensemble learning-based methods have been developed in recent years, such as TLCLnc [56], Simopoulos et al.’s work [125], and LncRNApred [111]. It was suggested that ensemble method likely obtains higher cross-species prediction performance. For example, TLCLnc achieved good performances on all 9 vertebrate species.

### 2.2 Features used in present computational tools

Feature selection is another vital factor for accuracy and specificity of prediction output. With the growing number of lncRNAs, features are gradually accumulated, from the earliest ORF length and coverage (CPC) [72], to conservative rating (phyloCSF) [84], to nucleotide composition (CNCI, PLEK) [80], [130], to structural features and epigenetic information (COME) [57]. These features include ORF length and coverage and integrity, nucleotide composition frequency such as GC content and k-mer scheme, codon usage and distribution, conservation scores such as substitution rate and phylogenic score, predicted RNA secondary structure, ribosome release score (RRS), epigenetic information, etc. Some features have several application limitations. For instance, features related to ORF and conservation score require assembly of full-length transcript for better performance [66]; calculation of the RRS relies on a well-defined ORF and 3′ untranslated region (UTR) [47]; epigenetics information is not provided extensively and species-specific [57]. Therefore, when establishing a model, it is important to choose the valuable features and remove redundancy in order to acquire an optimal outcome.

As far as the used features concerned, they can be refined into more categories according to the characteristics of the information they can provide, such as the nucleotide sequence-related, the secondary structure-related, the translational potential-related, the protein property-related, or the non-biological information-related, etc. In the process of feature selection, special attention should be paid to the issue of integrity, which includes “feature integrity” and “data integrity”. If the feature dimension is not complete, no matter how much data will not substantially improve the effect of the model, and *vice versa*. For most of the developed identification methods, they often adopted multiple features to optimize the accuracy and specificity of prediction results, because features with different natures probably have different contributions to the lncRNA identification. However, this does not mean that more features are always better, because “overfeaturing” will make the model to overestimate the impact of some aspects of the characteristics, which will significantly reduce model generalization and prediction performance. Moreover, too many features will render excessive expansion of vector dimensions, and thus increase computational complexity and running load. So, it is necessary to effectively select and combine the extracted features, so as to not only avoid redundancy but also improve model performance as much as possible.

### 2.3 The convenience of these tools

In addition, for the biologists with weak bioinformatics background, an important consideration to evaluate a computational tool is its availability, convenience, application scope and efficiency. Hence, we briefly evaluated the availability of current relatively-popular tools, on the aspects of soft-package download, webserver, data input format and dependency on reference genome (Table 2). For most commonly-used tools, they are inclined to adopt FASTA format as input, and some provide webserver interface, such as CPC2 and CNCI [72], [130]. Depending on the selected features, different methods show varied dependency on the reference genome. For instance, features such as conservation score (PhyloCSF and COME) [84], [57] and exon length (lncRScan-SVM) [129] heavily rely on a reference genome, resulting in limited application on non-model organisms lacking whole genome sequence or gene annotation. In addition, the difference of training datasets between methods can also influence prediction effect, thus some tools, such as PLEK, COME, LncADeep, provides model-retrain option for varied species [80], [57], [148].

**Table 2:** The availability of some commonly-used tools.

	Tools													
Availability		CPC2	PhyloCSF	CPAT	CNCI	iSeeRNA	PLEK	lncRScan-SVM	DeepLNC	COME	FEELnc	LncADeep	CPPred	LGC
Package	Online server	√		√	√	√								√
	Stand-alone	√	√	√	√	√	√	√	√	√	√	√	√	√

Applicable to	Model-retrain			√	√		√	√	√	√	√	√		
	Pre-built	√	√		√	√							√	√

Input format	FASTA	√	√	√	√		√		√		√	√	√	√
	BED			√		√								√
	GFF/GTF				√	√		√		√	√			√

Reference genome	-based		√				√	√		√				
	-free	√		√	√	√			√		√	√	√	√

The running time is also an important assessment factor for the application of tools, it depends on the adopted features and performance of models. As far as the reports by Li and his colleagues, PLEK runs faster, 8 times faster than CNCI, 244 times faster than CPC, and 1421 times faster than PhyloCSF [80]. In the work of COME, Hu and his colleagues compared the time cost of four tools, including COME, CNCI, RNAcode and HMMER; the order is COME > CNCI > HMMER > RNAcode [57]. In another work of Lncfinder, Han and his colleagues evaluated the speed of six tools, by using human data set that contains 2500 long non-coding transcripts and 2500 protein-coding transcripts. Their results showed that LncFinder (35.76 s), CPAT (9.05 s) and CPC2 (8.87 s) can predict several thousand sequences within 1 min and present reliable results. CNCI (1333.19 s) and PLEK (83.67 s) were slower. While CPC needed 4675.45 min to complete the process of alignment and identification. During the process of developing NCResNet, Yang and his colleagues estimated the running time of six models and got similar results. All six tools, NCResNet, CPC2, CPAT, IRSOM, LncFinder, and CPPred, are capable of large-scale (thousands to tens of thousands of sequences) lncRNA identification tasks [149].

## 3 Survey of the current *in-silico* tools of lncRNA identification according to selected features

As different lncRNA identification tools choose different machine learning algorithms and features, these tools have their own advantages and disadvantages for different types of noncoding RNA or experimental conditions. For all ncRNAs, they could be simply divided into two categories based on length threshold, small RNAs of length ≤ 200 bp (like miRNA, snRNA, piRNA, etc.) and lncRNAs of length >200 bp. The later can further be divided into many categories according to their location in genome, including intergenic lncRNA, sense/antisense lncRNA and intronic lncRNA.

For the early identification tools, they are not tailored for lncRNAs due to the inadequate recognition of lncRNAs; therefore, prediction of coding potential became a critical step for the subsequent lncRNA identification. One effective way is to compare unknown sequences with known protein data to detect the similarity between them, namely, the sequence conservation relative to encoding genes. These methods are often alignment-based, such as CONC, CPC and PhyloCSF. Certainly, characterization of coding potential has its own significance for genome annotation, so as to partition different functional regions on the genomes. Prodigal [60], TransDecoder [48], GeneMarkS-T [132] and CodAn [101] are such approaches that were developed for precise identification of coding regions in transcirpts, these methods have an important referential value for lncRNA identification. For example, using these tools, we can further determine the ORF-related features which were usually as a vital parameter during lncRNA identification.

Meanwhile, with the accumulation of knowledge about lncRNAs, more intrinsic features of lncRNAs were discovered, such k-mer frequency, the different secondary structure. In this way, some methods were developed specifically for lncRNA’s identification, such as LncRScan-SVM [129], lncRNA-MFDL [37], lncRNA-ID [1], lncRNApred [111], PLEK [80], CNCI [130], COME [57], DeepLNC [134]), FEELnc [146], etc. Some were even for a particular type of lncRNAs, such as linc-SF [142] and ISeeRNA [131], [128] that was designed for identification of intergenic lncRNAs. Next, we will respectively elaborate some methods according to the different attributes of features.

### 3.1 Alignment-based methods

Early identification tools tend to choose alignment-based methods due to the absence of systematic knowledge of lncRNAs. For these alignment-based methods, they heavily rely on the existence of known coding-gene sequences or databases. On the other hand, there are also some newly developed methods that need to align transcripts to genomes in order to integrate more genome-scale experimental data, such as expression profiles and histone modifications. Alignment-based methods may be limited when facing *de novo* sequencing of new organisms without well annotated genome sequences. In addition, due to the iterative alignments for searching homologous sequences, the alignment-based methods are extremely time-consuming when dealing with large-scale transcriptome data.

#### 3.1.1 Prediction based on primary sequence conservation

Researches had shown that the primary sequences of lncRNAs are poorly conserved. Therefore, the methods in this class are often used to perform BLASTX comparison with known protein databases to identify the encoded RNAs at first, and then screen out non-coding genes by eliminating the encoding genes in the transcriptomes. However, by analyzing the sequence similarity to known proteins or protein domains, it is likely to misclassify unknown coding transcripts into noncoding as false positive, thus requiring relatively high quality of known protein databases. As a result, to some non-model organisms, it is not friendly because of the shortage of well-established information on genome and transcriptome.

CPC is the representive of this kind of methods, which is based on SVM and adopts six features including three features based on ORF prediction and three features to conduct the alignments against UniProt proteins. The features based on ORF include log-odds score, ORF coverage and ORF integrity. Coding transcripts usually has a longer and more complete ORF with a higher log-odds score. The other three alignment-based features are hits number, hits score and frame score from BLASTX. Coding transcripts tend to have more hits with higher hits score and higher frame score [72]. As the earliest lncRNA identification tool, it is widely applied on lncRNA identification of many model organisms (such as human, mouse and Arabidopsis), with good performance. However, for many non-model plants, especially those species without well-established information of genome and transcriptome, its accuracy and specificity are reduced. Besides, the running speed of CPC was relatively low due to the process of pair-wised alignments. As reported in one study by Cabili and his colleagues, it took two days to identify the encoding capacity of 14,353 transcripts [18].

#### 3.1.2 Prediction based on phylogenetic analysis

Phylogenetic analysis screened lncRNAs from the perspective of species evolution, which employed the feature of codon substitution frequency (CSF) to discriminate lncRNAs from mRNAs [27]. One basic hypothesis about CSF is that the CSFs of ncRNAs between homologous species are different. Therefore, by aligning in multiple species to calculate the substitution frequency of codons of known mRNAs and ncRNAs respectively, we could obtain the different distributions of CSF scores for both mRNAs and ncRNAs in each species. It can be found that the CSF scores of mRNA or lncRNA have a completely different distribution.

PhyloCSF is such kind of method, which applied a comparative genomics method to assess the coding potential of nucleotide sequences by multiply aligning them with known protein-coding region across species and statistically analyzing phylogenetic codon models [84]. However, there are some defects of PhyloCSF. First, due to the poor conservation of lncRNA sequences, it is likely low efficient to seek out the homologs of lncRNAs between species [18]. Second, for those lncRNAs overlapping with the coding region, they are most likely to be mistaken for coding genes by PhyloCSF. In addition, multiple alignment takes a lot of time to perform comparison between species, therefore, the running speed of PhyloCSF software is relatively slower.

#### 3.1.3 Prediction according to secondary structure conservation

In term of the current knowledge on lncRNAs, they often function by binding with proteins, which needs these lncRNA molecules to hold a certain shapes or folds that are capable of conducting a variety of molecular functions [79], [99], [102], [144], [145], [122]. In this sense, the secondary structure of ncRNAs should be more conservative as compared with the primary sequence, because it likely harbors some important functional elements so as to specifically target proteins and genomic regions [94], [104], [73], [16]. However, it is not easy to assess the conservatism levels of secondary structure of ncRNA molecules; after all, for different ncRNA molecules, although their nucleotide sequences are completely different, they can still fold into the same structures, and thus perform the same functions. Taking the secondary structure of tRNAs as an example, that is, the sequence composition of tRNAs can be completely different but still have the same cloverleaf structure.

For the lncRNA molecules with longer length, exploring their structure conservation will be more difficult, since the prediction effect for the secondary structures of long sequences is not very good [40]and the functional structural regions are likely discrete. Hu and his colleagues had attempted to analyze the local structure conservation of lncRNAs by segmenting the long transcripts into shorter bins (100 bp), the later was used to calculate the RNA secondary structure conservation scores by scanning them against Rfam with the INFERNAL program (a binary score indicating the existence of a homologous structure in Rfam) [107]. As Hu and his colleagues found, the RNA secondary structure conservation features showed the highest specificity score, which meant most of the mRNAs had no conserved structures [57]. Thus, methods that incorporate lncRNA structural information are meaningful for an accurate identification of lncRNAs.

#### 3.1.4 Prediction according to genome-scale experimental features

It was proven that, as compared with mRNA, lncRNA also have other identifiable features which were found by means of genome-scale experiments, such as expression profiles, different types of histone modification, tissue specificity and ribosome release scores. For example, lncRNAs had relatively lower expression level, greater tissue specificity, and higher signals of H3K36me3 and H3K4me3 than mRNAs [33], [18]. Moreover, ribosome profiling data suggest that ribosomes may have divergent binding patterns on mRNAs and lncRNAs [47]. Therefore, these genome-scale experimental features could be used as the indicators to distinguish lncRNAs from mRNAs [42], [88], [89], [116], [43].

In the work of COME tool, Hu and his colleagues integrated multiple genome-scale experimental features, including expression profiles, histone modification, tissue specificity and the ribosome profiling features. It was found that adding these genome-scale experimental features could help to improve the prediction performance as well as the robustness between species [57]. However, obtaining these genome-scale experimental features is not easy. For example, the ribosome profiling features included ribosome release score (RRS) ([47], [137]) and translation efficiency score (TE) [62], [47]; but the calculation of TE and RRS scores required high expression levels for both mRNA and ribosome data, they were not available for most transcripts. In addition, how to integrate these genome-scale experimental features into the computational model is also a problem. COME used a two-step calculation procedure, which split the whole genome sequences into 100-nucleotide bins in the decompose step, and calculated the input features based on the indexed bins. Subsequently, in the compose step, they will use only three values (maximum, mean and variance) of all the bins for each feature vector of one transcript which usually have multiple bins [57].

### 3.2 Alignment-free methods

With the dramatically increased number of lncRNAs in recent years, the intrinsic differences of sequences between lncRNA and coding gene are extracted and explored for lncRNA identification. These features can be manifested at different levels of transcripts, including nucleotide primary sequences, translational potential of transcripts, RNA secondary structures, nucleotide/protein physicochemical characteristics, etc. Moreover, there are some methods that further transform/combine these basic features into high level features, such as structure parameters of complex network, which can be used for machine learning for distinguishing lncRNAs and mRNAs. Next, we will explain and illuminate them as followed.

#### 3.2.1 Features related to sequence intrinsic

This kind of features contain many contents, including the composition and arrangement of nucleic acid sequence (such as GC content, k-mer scheme, Fichett Score), codon use and neighborhood relationship (such as codon number, codon ratio, hexamer score), ORF-related features (such as ORF length, coverage and integrity), ect. The sequence compositions have demonstrated to be different between coding and noncoding; however, they varies from species to species, resulting in very unstable performances on different species [49]. ORF-related features including ORF length, ORF coverage and ORF integrity, are often used as the conventional evaluation criteria on the assumption of short-ORF RNAs having a low/no translational ability. K-mer scheme is a relatively robust feature for lncRNA identification, which represents the patterns of successive base sequences and have been adopted by many models, such as CNCI, PLEK, DeepLNC [130], [80], [134]. Hexamer score is simplified k-mer scheme, which fixes K = 6 so as to evaluate the neighborhood relationship between two adjacent codons, such as CPAT [141].

CPAT is an alignment-free lncRNA identification tool, which applied four features to identify lncRNA, including the longest ORF length, ORF coverage, Fickett score, hexamer score. Among them, Fickett score and hexamer score each can be used as a classifier of ncRNA [38]. Fickett score calculates the preference and composition frequency of A, C, G and T bases in codon, while hexamer score calculates the combination frequency of six bases (hexamer) of adjacent amino acids in transcript sequences. Leaning upon the intrinsic divergence between lncRNA and coding gene, CPAT used logistic regression to construct the classification model [141].

The CNCI method also evaluates the coding capability of transcript sequences according to nucleotide usage frequency with SVM. CNCI introduces a concept of ANT (adjoining nucleotide triplets), which is similar to the hexamer of CPAT. Firstly, CNCI constructs two ANT Score Matrix to evaluate the usage frequency of all kinds of ANTs in coding and noncoding genes respectively. For each candidate transcript, CNCI uses a sliding window strategy by a step length of three nucleotides to generate six reading frames, and calculates the sequence-score (S-score) of each frame based on ANT score matrix. By producing six discrete numerical arrays, the most likely coding domain sequence is identified [130]. CNCI has a good performance for poorly annotated species or those without whole-genome sequence information, but it may misclassify transcripts that contain insertion or deletion (indel) sequencing errors [80].

The PLEK method was developed for distinguishing lncRNAs from coding RNAs, based on an improved k-mer scheme and a SVM algorithm. The k-mer parameters in PLEK range from 1 to 5. By adopting a sliding-window strategy with a step length of one nucleotide, PLEK counts the occurrence number of all kinds of k-mer strings in each transcript, and exploits the calibrated k-mer usage frequencies of each transcript as computation features [80]. DeepLNC also used k-mer scheme as features. The difference is that DeepLNC uses the traditional k-mer scheme as a sole feature, the k values selected in DeepLNC are 2, 3, and 5 [134].

#### 3.2.2 Features related to transcript’s coding potential

The features under this category are associated with the coding potential of transcripts, and thus are likely confusing with ORFs. Since ORFs are the conceivable coding sequences predicted by reading frame, here, we designate these transcript-related features as ones supported with more translatable evidences, such as ribosome binding and release scores.

During protein translation, the ribosomes interact with mRNAs to initiate translation and finally release from mRNAs to terminate translation [124]. Based on this fact, Achawanantakun and his colleagues developed an lncRNA identification tool named lncRNA-ID, which integrated ribosome interaction features that involved various stages of translation. There are two features from Kazak motif for translation initiation, three features against ribosome coverages on three regions (the whole transcript, ORF and 3′UTR) for translation process, and ribosome release score (RRS) to capture the translation termination signal [1]. The results showed that combination of multiple groups of features leads to better performance than using a single group of features, and the ribosome interaction features present the best discriminative power [1].

Recently, the combined feature of composition, transition, and distribution (CTD) was found to be associated with the coding potential of RNA transcripts. The composition features mean the frequency of amino acids with a particular trait in the total theoretically translated products; the transition features reflect the variation trend of two adjacent amino acids; while the distribution features are to assess the position and distribution of amino acids with a certain property. According to the results of NCResNet and CPPred models, CTD features are valuable in predicting RNA coding potential, especially for sORF data, and thus, to improve the performance on sORF data significantly [149], [133].

#### 3.2.3 Features related to RNA secondary structure

For lncRNAs, their secondary structures probably has more important roles for biological functions, therefore, relatively more conservative than mRNAs [17], [95]. To some extent, the sequence-derived features of lncRNAs present the surface content of nucleotide strings, whereas the secondary structure features may imply some important functional information.

To explore the discriminating power of this category, lncRNA-MFDL constructed a deep learning model by fusing the secondary structure with ORFs, k-mer and the most-like coding domain sequences to discriminate lncRNAs and mRNAs [37]. LncFinder introduced multi-scale secondary structural features at three levels: stability, secondary structure elements combined with pairing condition and structure-nucleotide sequences [50]. The minimum free energy (MFE) scores were used to evaluate the secondary structure stability. Generally, lncRNAs are less stable than mRNAs [28], with a lower MFE. It was found that secondary structural features surpassed features of transcript length, Fickett score and pI (isoelectric point) value, demonstrating a considerable discriminating power of structural features [50].

However, the use of secondary structure features alone is not statistically robust enough to detect lncRNAs. This is because a random RNA with low GC content can also fold into low-energy structure. Besides, in term of the importance of RNA secondary structure on biological function, we can exploit the features of secondary structure to further sub-classify the internal functions of ncRNAs. For example, Childs and his colleagues developed a method, named GraPPLE, for classifying non-coding RNA molecules as functional and, furthermore, into Rfam families based on the graph properties of the predicted RNA secondary structure. By graphical RNA molecules, both local–global and global structural properties are captured, which can be used to further deduce the large- and small-scale structural as well as functional differences between molecules. Thus, GraPPLE may provide a valuable computational tool to discover potentially interesting RNA molecules among large candidate datasets [23].

#### 3.2.4 Features based on physicochemical property of nucleotide/proteins sequences

Several tools applied physicochemical properties of nucleotide/proteins sequences as features, such as pI values of predicted proteins in CPC2 and CPPred, electron–ion interaction pseudo-potential (EIIP) of nucleotide sequences in LncFinder and NCResNet.

CPC2 is the update of CPC, and also uses SVM to construct classifier, but no need for alignment. It mainly integrated four features: the longest ORF length, ORF integrity, Fickett score, and pI value [68]. It was assumed that the peptides artificially identified in a non-coding transcript should have different chemical properties when compared with these real ones encoded by coding sequences. The characteristic of pI is obtained by translating the longest ORF into amino acid sequence and then calculating the physicochemical property of pI of amino acid [12]. As a result, pI feature obtained good performance in CPC2 model. In another work, CPPred also used pI as a feature, it was found that pI feature is human-specific [133].

The use of pI is trying to theoretically transform RNA sequence into protein sequence. In the work of LncFinder, Han and his colleagues explored the physicochemical property of nucleotide sequence, EIIP, as feature. EIIP was initially used to indicate the power spectrum distribution for the coding region of transcripts, which are totally different from ncRNAs [103]. For any DNA sequence, nucleotides can be converted into different EIIP values: A → 0:1260; C → 0:1340; G → 0:0806; T → 0:1335 [103]. Compared with pI values, EIIP values are directly from RNA sequences, thus avoiding the potential bias caused by the speculated translation process [50].

#### 3.2.5 Features derived from transformation/combination

The features in the data directly affect the prediction model you use and the results you can achieve. So far, in order to distinguish lncRNAs from coding genes, many features have been selected. In addition to *de novo* extraction, new features can also be obtained in other ways, such as reanalysis of current known features, or combination of different types of features. It is very attractive to get new features through transformation/combination, which often means that the model is more concise and the prediction performance is better. In addition, obtaining new features through transformation/combination also allows us to learn more about the nature of the prediction problems, although sometimes feature transformation/ combination implies a higher level of abstraction.

For instance, in the work of Tripathi and his colleagues, traditional k-mer features has been further transformed into the form of entropy [134]. In the proposed Deep Neural Network model (DeepLNC), the k-mer information has been used as a sole feature, and generated on the basis of Shannon entropy function, which resulted in improved classifier accuracy. Another interesting example is about BASiNET, an alignment-free lncRNA identification tool based on the feature extraction from complex network measurements [64]. Using the concept of complex network, BASiNET transformed the k-mer information extracted from the sequence into an undirected weighted network, in which the nodes represent the words (k-mers), and the weight of an edge represents the frequency that one word was identified as a neighbor from another word. Furthermore, this method applied a threshold to the weight of the edges in order to view different resolutions of the network, and used a couple of network topological measures as new features.

These features, entropy used in DeepLNC or network structure parameters used in BASiNET, are high level features transformed/combined from basic features. The acquisition of these features does not require prior biological information, such as genome annotation or homologous sequence alignment. But on the other hand, these biological-information-free features contain a lot of hidden biological significance. Whether it is the different distribution trend of various k-mers, or the most persistent edges (patterns) in the BASiNET network, they are worthy of further exploration.

## 4 Challenges and future perspectives

### 4.1 New data and new features

In order to efficiently characterize lncRNAs from coding RNAs, researchers have been engaged in improving algorithm models and features. In most cases, a dramatic advance on algorithm is not practical in a short term. Hence, more attention was paid for acquiring new data and new features, which can be optimized by either deep understanding of lncRNA properties or technological progress. Until now, many features were selected for distinguishing lncRNAs from coding genes, they could work as a single or as combined feature sets, with different scopes of application. Given that feature extraction is sensitive to small perturbation of the training dataset, the prediction capability of each method is likely skewed in accuracy and specificity, especially when facing *de novo* assembling transcriptome data with no high-quality genome annotation. Therefore, features with greater commonness would facilitate lncRNA identification across species, such as the k-mer scheme of PLEK, TLCLnc and IRSOM [80], [56], [112], ORF length of CPAT and CPPred [141], [133] and GC content of COME and LGC [57], [139].

In terms of data types, previous studies on lncRNAs mainly focused on species of animals, while there was relatively little discussion on plants. With the increased transcriptome data of plant samples, the functional cognition for plant lncRNAs is becoming more and more important. Therefore, some methods are developed specifically for identification of lncRNAs in plants, such as RNAplonc [108], CREMA [125]and PLIT [32]. The replenishment of plant data increases the diversity of lncRNA sequences, prevents the data from animal bias, and is conducive to optimizing the extraction of lncRNA features. On the other hand, as the plant genomes have experienced a lot of duplication, especially at the whole genome level, it is likely to exist a lot of paralogs of lncRNAs. This fact can further promote the analysis of lncRNA evolution to some extent, and facilitate to find more conservative function domains or motifs, which will ultimately help lncRNA function prediction. Meanwhile, these increasing data of lncRNAs in plants provides a reference pool in order to deeply evaluate how the features really perform on the lncRNA identification.

Since lncRNAs were not well understood in the early stage, some simple features involved in coding potential, such as ORF, were used to screen lncRNAs, but they could not distinguish lncRNAs from other types of ncRNAs. One simple criterion for determining whether a transcript is a lncRNA is to set the length threthold of greater than 200nt. However, with the biological significance increase of lncRNAs, there emerged some specific methods for identification of lncRNAs, such as COME considering the unique epigenetic information and secondary structure conservation of lncRNAs [57]. Meanwhile, some new features have also been proposed, such as entropy and network structure parameters, all of which appeared to have a relatively high relevance with lncRNA identification [134], [64]. New features can be discovered in several ways: *de novo* extraction, reanalysis of current known features, or combination of different types of features. For instance, k-mer information can be further converted into the form of entropy [134]. These new features can not only help to identify coding/noncoding genes, but also further subdivide each category internally. For example, Grapple employed graph theory model to further perform the functional classification within ncRNAs [23].

### 4.2 The discovery of bifunctional RNA blurred the boundary between coding and noncoding

The past knowledge on lncRNAs is non/low protein coding [52]. Therefore, classification of genes into coding or non-coding often depends on whether the transcript holds a long, or even conserved ORF, and this length cutoff often sets as 300 nts for most lncRNA identification tools. However, increasing evidence demonstrated that lncRNAs in various eukaryotic organisms harbor sORFs and can express functional micropeptides with length less than 100 amino acids [82], [5], [34], [41], [51], [58], [83], [91]. Studies on lncRNA-encoded functional micropeptides in eukaryotes were initially found in plant [85], [67]. The early nodulin 40 (Enod40) gene in legume, previously annotated as lncRNA, encodes two peptides of 12 and 24 AA residues which regulate root nodule organogenesis by binding with a sucrose synthesizing enzyme [119]. Three other micropetides, Brick1 (Brk1) in maize, POLARIS (PLS) and ROTUNDIFOLIA (ROT4) in Arabidopsis, were found to be involved in leaf morphogenesis [24], [39], [106]. Another micropeptide, kiss of death (KOD, 25 AAs) in Arabidopsis, acts as an inducer of programmed cell death [13]. In animal, lncRNA-derived sORFs displayed more abundant diversity on biological functions. The micropeptides, MLN, Scl and MOTS-C in human can regulate the activities of SERCA (sacro/endoplasmic reticulum Ca^2+^-ATPase) in the muscle-specific tissues [78], [91]. AGD3 encodes a small protein of 63 AAs that modulates human stem cell differentiation [70]. The polished rice or tarsal-less (tal) gene in *Drosophila* encodes four micropeptides from 11 to 32 AAs, all of which play a vital role in tarsal morphogenesis in the fly leg [41]. All these facts imply that sORFs-encoded micropeptides originated from noncoding regions are capable to exert important regulatory roles in fundamental biological processes, and have been oversighted previously because of their small size. Some large-scale experimental approaches developed in recent years, such as ribosome profiling sequencing (*ribo*-seq) [61], [62]and mass spectrometry (MS) [9], [127], further promote the discovery of sORF-encoded peptides, unraveling that translation is more extensive than initially thought. By far, there were thousands of translated sORFs discovered in lncRNAs in various species [63], [11], [120], [65], some of which are translated as frequently as canonical protein-coding ORFs or well conserved across species [7], [115], suggesting the potential functionality of these sORFs.

On the other hand, studies showed that a protein-coding RNA can also perform non-coding functions. For example, independent of the tumor suppressor function on the form of protein, p53 gene encoded a triple synonymous mutant (TriMp53) in codons, which has an increasing affinity for Mdm2 (an E3 ubiquitin-protein ligase), thus *in-cis* suppressing p53/TP53 protein ubiquitination [20], [19]. The ASCC3 gene encodes a helicase involved in DNA repair, which could be switched into a shorter lncRNA by UV-induced alternative splicing [14], [143]. Protein Phosphatase 1 Nuclear Targeting Subunit (PNUTS or PPP1R10) was originally designated as a protein-coding gene encoding an inhibitory regulatory subunit of protein phosphatase-1 (PP1) [3]. It can dynamically switch into LncRNA-PNUTS in the effect of actinomycin-D and cycloheximide. LncRNA-PNUTS was supposed to regulate epithelial-to-mesenchymal transition (EMT) and cell migration as a competing endogenous RNA (ceRNA) for miR-205, a primary regulator of EMT-related transcription factors [45], [76]. The facts that lncRNAs harbor sORF and mRNAs also express non-coding transcript variants blur the boundary between coding and noncoding genes, posing a further challenge on the identification of gene coding potential [71], [105], [82].

### 4.3 The dilemma of current tools on sORF-contained lncRNAs

However, currently-developed computational methods often have a poor performance on sORF-contained lncRNAs, since most of them integrated ORF-related features (ORF length, ORF coverage, ORF integrity) for analyzing [97], [81], [133]. As compared with canonical protein-coding ORFs, sORFs derived from lncRNAs are difficult to acquire statistically significant values because of the very short length of the sequences and the low number of possible changes [92], [74]. Consequently, a number of RNA molecules have been designated as non-coding and actually harbor short open reading frames (sORFs) that code for functional peptides, which have been omitted due to their small size. Indeed, some work had concerned about this problem recently. Tong and coworkers developed CPPred model to improve the prediction performance on sORF data [133], by introducing CTD features that are associated with the process of protein translation by integrating the information of nucleotide composition, nucleotide transition and nucleotide distribution [36]. Additionally, several merits, such as methylation, ribosome release score (RRS) that detects the translation termination at the stop codon at the end of an ORF [137], [47], additional structural elements like internal ribosome-entry sites (IRES) [53], [35], [110], [150], were take account into detecting the potential sORFs in transcripts.

We also attempted to analyze the divergence between ORFs stemming from coding regions and that from noncoding regions by using our in-home python script. We firstly integrated a set of lncRNA-encoded amino acid sequences, which were obtained from CNC database (http://www.rna-society.org/cncrnadb/) and have been verified by human experiments or found by mass spectrometry; then, we downloaded all human protein-coding sequences from Gencode V34. We compared the length and amino acid composition of mRNA and lncRNA ORFs. Our results show that lncRNA-encoded amino acid sequences are significantly shorter than ordinary protein sequences, which is an obvious result, and there are also significant differences of k-mer distribution between their amino acid sequences, which is an interesting result (data not show).

**Fig. 1:**
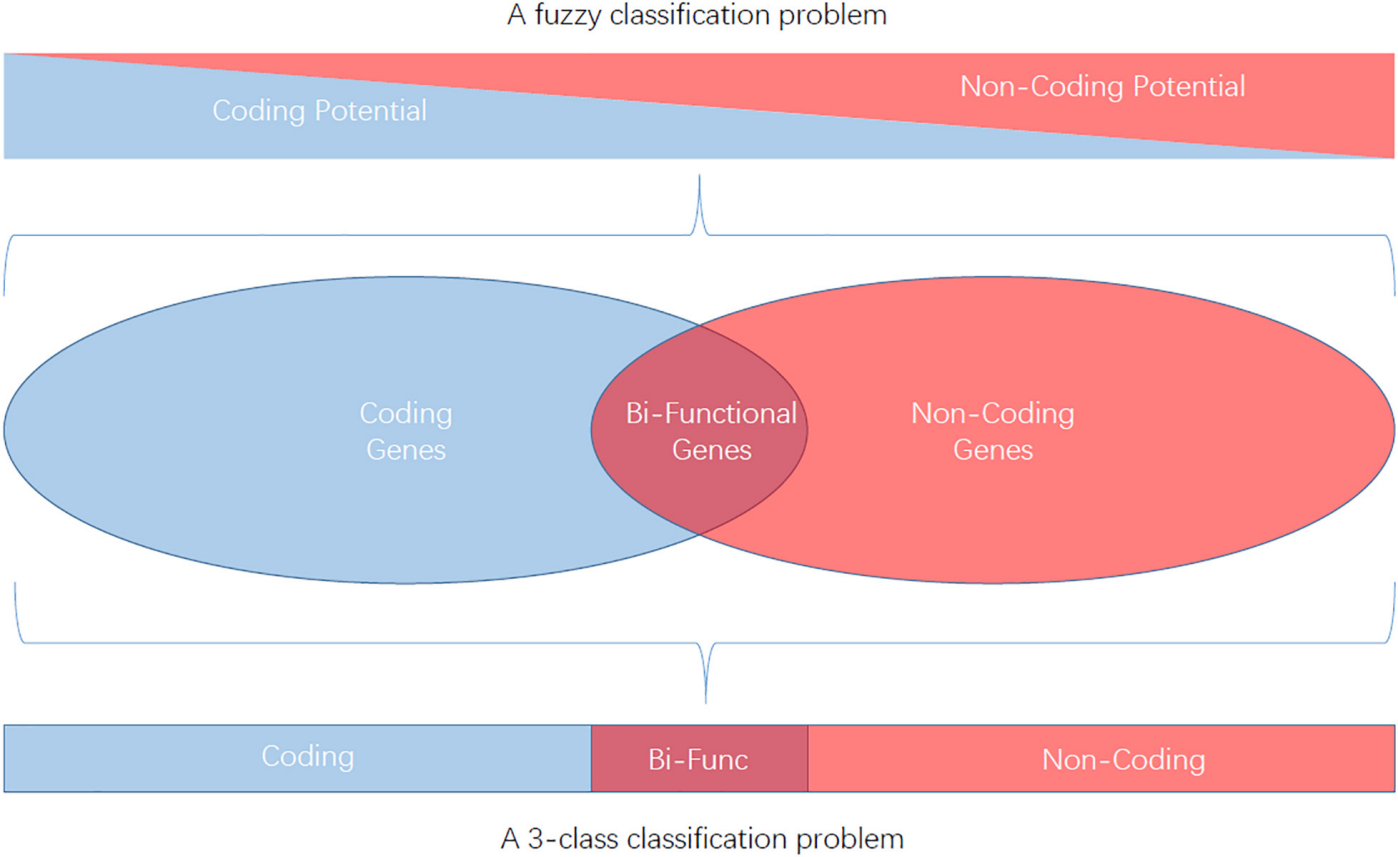
A possible fuzzy or multiple classification model for lncRNA identification.

All these facts raised the questions that whether we should dynamically look upon the concept of coding potential in the view of evolutionary significance, or whether it is suitable to use current dichotomy classifiers for these “coding and noncoding” bifunctional or hybrid genes. In the future, it is necessary to consider how to integrate these new high-throughput data more effectively, such as *ribo*-seq and high resolution MS. Therefore, collecting more manually curated data and extensive data exploring are on an urgent demand. On the other hand, we need to develop a better classification model for bifunctional RNA. First, we should investigate the prediction results for bifunctional RNAs of the current tools that are developed based on binary classification model, and whether these tools based on different models and features have different preferences. Further, we should consider whether we need to introduce other classifier model, such as multiple classifiers systems, or fuzzy classification (Fig. 1). Compared to multiple classifiers system, we think that fuzzy classification maybe a better choice. Fuzzy classification is the process of grouping elements into a fuzzy set, which is a mathematics term and remarks some sets whose elements have degrees of membership [153], [31]. All these problem need to be discussed and solved in the future. We hope this review could bring new thinking and inspiration on this field.

## CRediT authorship contribution statement

**Jing Li:** Conceptualization, Data curation, Investigation, Writing - original draft, Writing - review & editing. **Xuan Zhang:** Data curation, Software, Writing - review & editing. **Changning Liu:** Conceptualization, Funding acquisition, Supervision, Writing - original draft, Writing - review & editing.

## Declaration of Competing Interest

The authors declare that they have no known competing financial interests or personal relationships that could have appeared to influence the work reported in this paper.
